# Socioeconomic Deprivation, Primary Care Physician Workload and Colorectal Cancer Screening Adherence Across Italian Regions: An Ecological Spatial Analysis

**DOI:** 10.3390/medsci14040436

**Published:** 2026-07-26

**Authors:** Caterina Elisabetta Rizzo, Vincenzo Restivo, Maria Concetta Rizzo, Cristina Genovese

**Affiliations:** 1 Department of Prevention, Local Health Authority of Enna, 94100 Enna, Italy; 2 Department of Medicine and Surgery, University of Enna “Kore”, 94100 Enna, Italy; vincenzo.restivo@unikore.it; 3 Department of Political and Social Sciences, University of Catania, 95100 Catania, Italy; maria.concetta.2000@hotmail.it; 4 Department of Biomedical Sciences, Dental Sciences and Morpho-functional Imaging, University of Messina, 98100 Messina, Italy

**Keywords:** colorectal cancer screening, primary care, socioeconomic deprivation, cancer prevention, health inequalities, real-world data

## Abstract

**Background:** Socioeconomic deprivation is a well-established determinant of participation in colorectal cancer (CRC) screening. However, less is known about the geographic clustering of screening adherence and whether spatial inequalities mirror regional patterns of socioeconomic disadvantage. This study investigated the association between socioeconomic deprivation and adherence to CRC screening across Italian regions and explored the presence of spatial autocorrelation using geographic analytical methods. **Methods:** We conducted a nationwide ecological cross-sectional study including 21 Italian regional units. Pearson correlation analysis was performed to explore associations between colorectal cancer (CRC) screening adherence, primary care physician (PCP) workload and socioeconomic deprivation. Multivariable linear regression was used after assessment of multicollinearity using variance inflation factors (VIFs). Sensitivity analyses included alternative deprivation indicators and leave-one-region-out analyses. Spatial dependence was evaluated using Global Moran’s I and Local Indicators of Spatial Association (LISA). **Results:** CRC screening adherence showed substantial geographic variability across Italy, with higher participation in northern regions and lower participation in southern areas. Screening adherence was inversely associated with socioeconomic deprivation. Sensitivity analyses confirmed the robustness of the association across alternative deprivation measures and sequential exclusion of each regional unit. Spatial analysis demonstrated significant positive spatial autocorrelation (Moran’s I = 0.275; *p* = 0.039), indicating that neighbouring regions tended to exhibit similar screening behaviours. LISA analysis identified significant High–High clusters in northern Italy, Low–Low clusters in southern and insular regions, and isolated spatial outliers, highlighting persistent territorial inequalities in preventive healthcare utilisation. **Conclusions:** Regional disparities in CRC screening participation were associated with socioeconomic deprivation and modest but significant spatial clustering. These findings provide a regional-level description of inequalities in screening participation and may help inform future public health strategies aimed at reducing disparities in organised colorectal cancer screening.

## 1. Introduction

Colorectal cancer (CRC) remains one of the leading causes of cancer-related morbidity and mortality worldwide, despite being one of the few malignancies for which population-based screening has consistently demonstrated a reduction in both incidence and mortality through the detection and removal of premalignant lesions and early-stage cancers [1]. In Europe, organised screening programmes based on the faecal immunochemical test (FIT) have become a cornerstone of cancer prevention policies, yet participation rates remain highly heterogeneous across and within countries [2].

Italy has implemented organised CRC screening nationwide through its National Health Service [3], but marked regional inequalities persist. Northern regions generally achieve substantially higher participation rates than southern regions, resulting in unequal opportunities for early diagnosis and prevention [4]. Previous studies have consistently shown that socioeconomic disadvantage—including poverty, lower educational attainment, limited health literacy, and social exclusion—is associated with lower participation in cancer screening programmes [5]. These disparities represent an important challenge for equitable cancer prevention within universal healthcare systems.

Beyond individual socioeconomic factors, increasing attention has been directed towards the organisational capacity of healthcare systems to sustain preventive services [6]. Primary Care Physicians (PCPs) play a pivotal role in promoting CRC screening through patient counselling, reinforcement of screening invitations, and facilitation of follow-up investigations [7]. However, many European healthcare systems, including Italy, are experiencing a progressive shortage of PCPs accompanied by increasing patient-to-physician ratios [8]. Such workforce pressures may reduce the time available for preventive activities, although their population-level impact on organised screening programmes remains poorly understood [9].

At the same time, advances in digital health have created new opportunities to support organised cancer screening independently of traditional physician-centred models [10]. Electronic recall systems, behavioural nudges, patient portals, and mobile health applications can improve participation while reducing the administrative burden on primary care services [11]. Nevertheless, the effectiveness and cost-effectiveness of these interventions are likely to depend on the local context, particularly the coexistence of healthcare workforce constraints and socioeconomic disadvantage [12]. Identifying the regions in which structural barriers have the greatest impact may therefore help prioritise future digital oncology strategies.

Understanding the relative contribution of these factors is essential for informing public health policies aimed at reducing inequalities in cancer prevention. The present study aimed to evaluate the associations of primary care workload and regional socioeconomic deprivation with organised CRC screening adherence across Italy. We hypothesised that socioeconomic deprivation would explain a greater proportion of regional variation in screening participation than primary care workforce capacity. We also investigated whether screening adherence exhibited significant global and local spatial clustering.

## 2. Materials and Methods

### 2.1. Study Design

A nationwide ecological study was conducted to investigate the association between primary care workforce capacity, socioeconomic deprivation and adherence to the organised colorectal cancer (CRC) screening programme across Italy. It was conducted using aggregated data from 21 Italian regional units, comprising 19 Regions and the Autonomous Provinces of Trento and Bolzano. For spatial analyses, Trento and Bolzano were combined into the single territorial unit of Trentino-Alto Adige, resulting in 20 geographic units. The study was based exclusively on aggregated regional data from publicly available institutional databases and is reported in accordance with the Strengthening the Reporting of Observational Studies in Epidemiology (STROBE) recommendations for observational studies [13].

### 2.2. Data Sources

Data referred to the 2024–2025 organised CRC screening cycle and were obtained from three national institutional sources.

Regional CRC screening adherence rates were retrieved from the Italian National Screening Observatory (Osservatorio Nazionale Screening, ONS）, which annually monitors the performance of organised cancer screening programmes nationwide [14].

Information on primary care workforce capacity, including the number of active Primary Care Physicians (PCPs) and the resident population in each region, was obtained from the National Agency for Regional Health Services (AGENAS) [15]. Regional socioeconomic indicators were extracted from the Italian National Institute of Statistics (ISTAT) [16].

Because the study analysed anonymised aggregated data available in the public domain, neither informed consent nor approval from an Institutional Review Board was required according to current Italian regulations governing observational ecological research.

### 2.3. Study Variables

As described in Table 1, the primary outcome was regional adherence to the organised colorectal cancer screening programme, expressed as the percentage of invited individuals aged 50–69 years who completed a faecal immunochemical test (FIT) during the reference screening round. Primary care workforce capacity was assessed using the resident-to-primary care physician ratio, calculated by dividing the resident population by the number of active PCPs in each region.

Higher values indicate greater physician workload and reduced theoretical availability of time for preventive clinical activities. Regional socioeconomic conditions were characterised using four deprivation indicators published by ISTAT for 2025 [16].

Because these indicators measure related dimensions of socioeconomic disadvantage and exhibit substantial collinearity, all were initially explored through univariable correlation analyses with CRC screening adherence. The population at risk of poverty or social exclusion was subsequently selected as the principal socioeconomic covariate for multivariable modelling because it demonstrated the strongest inverse association with screening adherence and represents the most comprehensive indicator of structural social vulnerability adopted by Eurostat and ISTAT [17].

### 2.4. Statistical and Spatial Analysis

Continuous variables are presented as mean ± standard deviation (SD), whereas categorical variables are reported as frequencies and percentages. Descriptive analyses summarised regional variation in colorectal cancer (CRC) screening adherence, primary care workforce capacity, and socioeconomic indicators across Italy.

Unavailable values (reported as NA in Table 1) reflected indicators not released by ISTAT for specific regions rather than missing study data. Pearson correlation analyses were conducted using complete-case analysis for each deprivation indicator. No missing values were present for the variables included in the final multivariable regression model.

Pearson correlation coefficients were calculated to evaluate the univariable association between CRC screening adherence and each explanatory variable. Variables demonstrating the strongest epidemiological relevance and univariable association with the outcome were subsequently entered into a multivariable linear regression model to estimate the independent association between primary care workload and CRC screening adherence after adjustment for socioeconomic deprivation. Regression coefficients (*β*), 95% confidence intervals (95% CI), standard errors (SE), t-statistics and corresponding *p*-values were reported.

Model performance was assessed using the coefficient of determination (*R*^2^) and adjusted *R*^2^. Regression assumptions were evaluated through inspection of residual plots, the Shapiro–Wilk test for residual normality, the Breusch–Pagan test for homoscedasticity and the Variance Inflation Factor (VIF) to assess multicollinearity.

Prior to multivariable modelling, pairwise Pearson correlation coefficients were calculated among all candidate socioeconomic indicators to assess the degree of intercorrelation (Supplementary Table S1). Variance inflation factors (VIFs) were subsequently estimated using an exploratory model including all candidate predictors to evaluate multicollinearity. Because the socioeconomic deprivation indicators showed substantial collinearity, only the composite At Risk of Poverty or Social Exclusion (AROPE) indicator was retained in the final multivariable model (Supplementary Table S2). To assess the robustness of the findings, sensitivity analyses were performed by replacing the AROPE indicator with each of the remaining socioeconomic deprivation indicators (population at risk of poverty, severe material and social deprivation, and low work intensity), while maintaining the resident-to-primary care physician ratio in all models (Supplementary Table S3). To evaluate model robustness, a leave-one-region-out sensitivity analysis was performed by repeating the primary multivariable regression after sequential exclusion of each regional unit.

To investigate whether CRC screening adherence exhibited geographical spatial dependence, a spatial autocorrelation analysis was additionally performed. For spatial analyses, the Autonomous Provinces of Trento and Bolzano were combined into Trentino-Alto Adige, resulting in 20 territorial polygons. A first-order Queen contiguity spatial weights matrix was constructed using shared boundaries or vertices and row-standardised before analysis. Sicily and Sardinia were retained as disconnected island observations, and no artificial ferry or cross-sea connections were introduced. Regional adjacency was defined using a first-order Queen contiguity spatial weights matrix [18]. Global spatial autocorrelation was assessed using Moran’s I statistic with 9999 Monte Carlo permutations. To assess whether residual spatial dependence remained after adjustment, Global Moran’s I was also calculated for the residuals of the OLS model fitted to the spatially harmonised 20-region dataset. The absence of significant residual autocorrelation was considered supportive of the adequacy of the non-spatial OLS specification. Local Indicators of Spatial Association (LISA) were subsequently calculated to identify regional clusters of high and low screening adherence [19]. Regions were classified into High–High, Low–Low, High–Low, Low–High and non-significant clusters according to Local Moran statistics (*p* < 0.05). The contiguity-based spatial matrix left Sicily and Sardinia without terrestrial neighbours. Although no arbitrary connections were introduced, local spatial statistics for island regions should therefore be interpreted cautiously.

Statistical and regression analyses were performed using R version 4.6.0 [20]. Spatial autocorrelation analyses, including the computation of Global Moran’s I and Local Indicators of Spatial Association (LISA), were executed within the Google Colab cloud environment using Python version 3.10 (incorporating GeoPandas v0.14.1, libpysal v4.8.0, and esda v2.5.1 libraries). Finally, thematic choropleth maps and the final visualization of spatial clusters were generated using QGIS (Quantum Geographic Information System) version 4.0.3 [21]. Statistical significance was defined as *p* < 0.05.

## 3. Results

### 3.1. Regional Characteristics

The analysis included all 19 Italian Regions and the 2 Autonomous Provinces (*n* = 21). Marked geographical variability was observed in organised colorectal cancer (CRC) screening adherence, primary care workforce capacity, and socioeconomic conditions across Italy.

**Figure 1:**
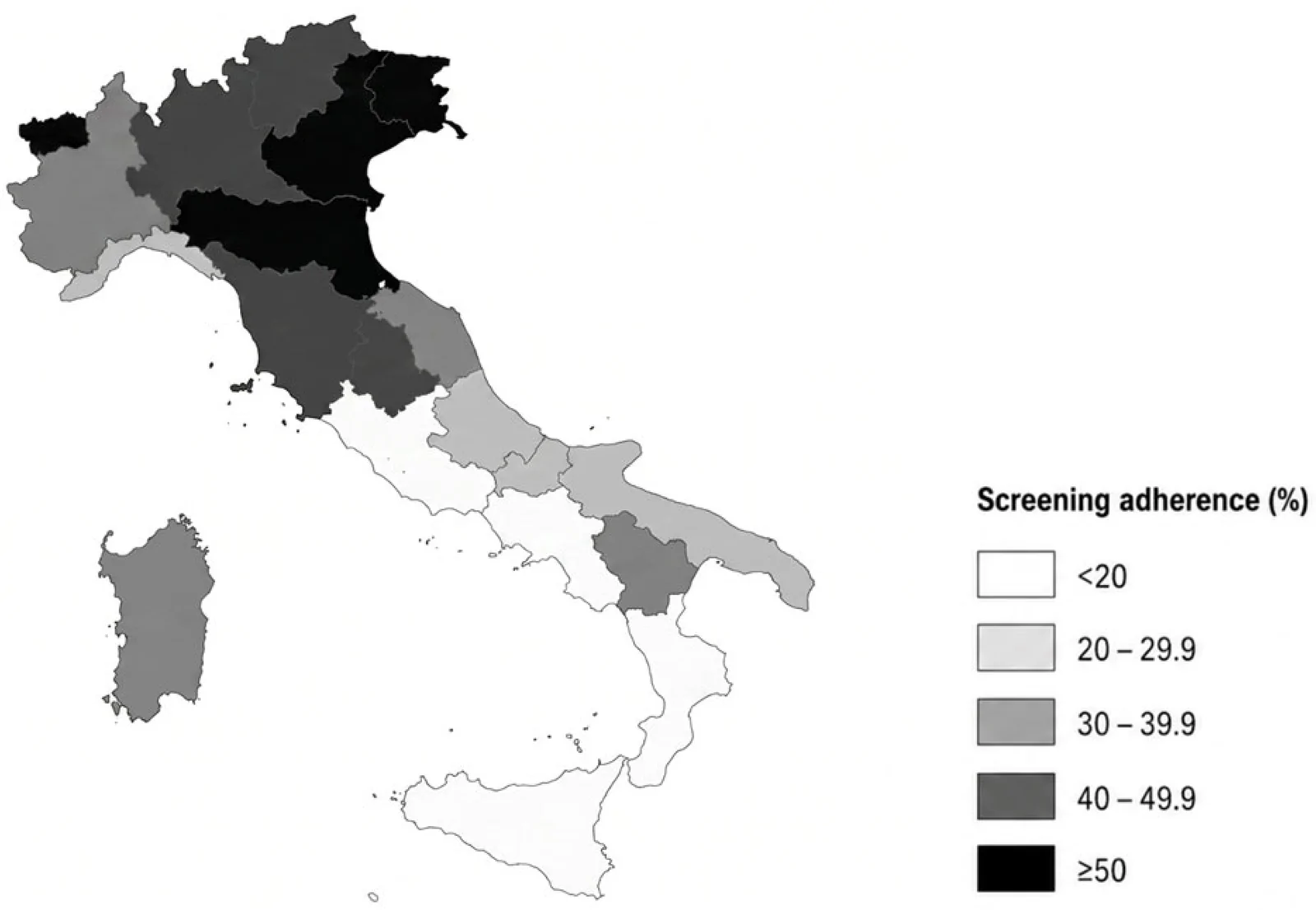
Geographic distribution of organised colorectal cancer screening adherence across Italy.

Regional screening adherence ranged from 5.2% in Calabria to 66.4% in Valle d’Aosta, whereas the resident-to-primary care physician (PCP) ratio ranged from 1169 residents per PCP in Basilicata to 1595 residents per PCP in Lombardia. Similarly, the proportion of the population at risk of poverty or social exclusion ranged from 5.6% in Valle d’Aosta to 45.3% in Calabria, highlighting the pronounced socioeconomic gradient between northern and southern regions. Detailed regional characteristics are reported in Table 2. Also, as shown in Figure 1, the spatial distribution of colorectal cancer screening adherence highlights a clear North–South divide in Italy.

Summary statistics for the study variables are presented in Table 2. Mean CRC screening adherence across Italy was 35.9% ± 15.1%, while the mean primary care workload was 1397 ± 133 residents per PCP (Table 3). The mean proportion of the population at risk of poverty or social exclusion was 21.0% ± 12.0%.

### 3.2. Multivariable Linear Regression Analysis

**Figure 2:**
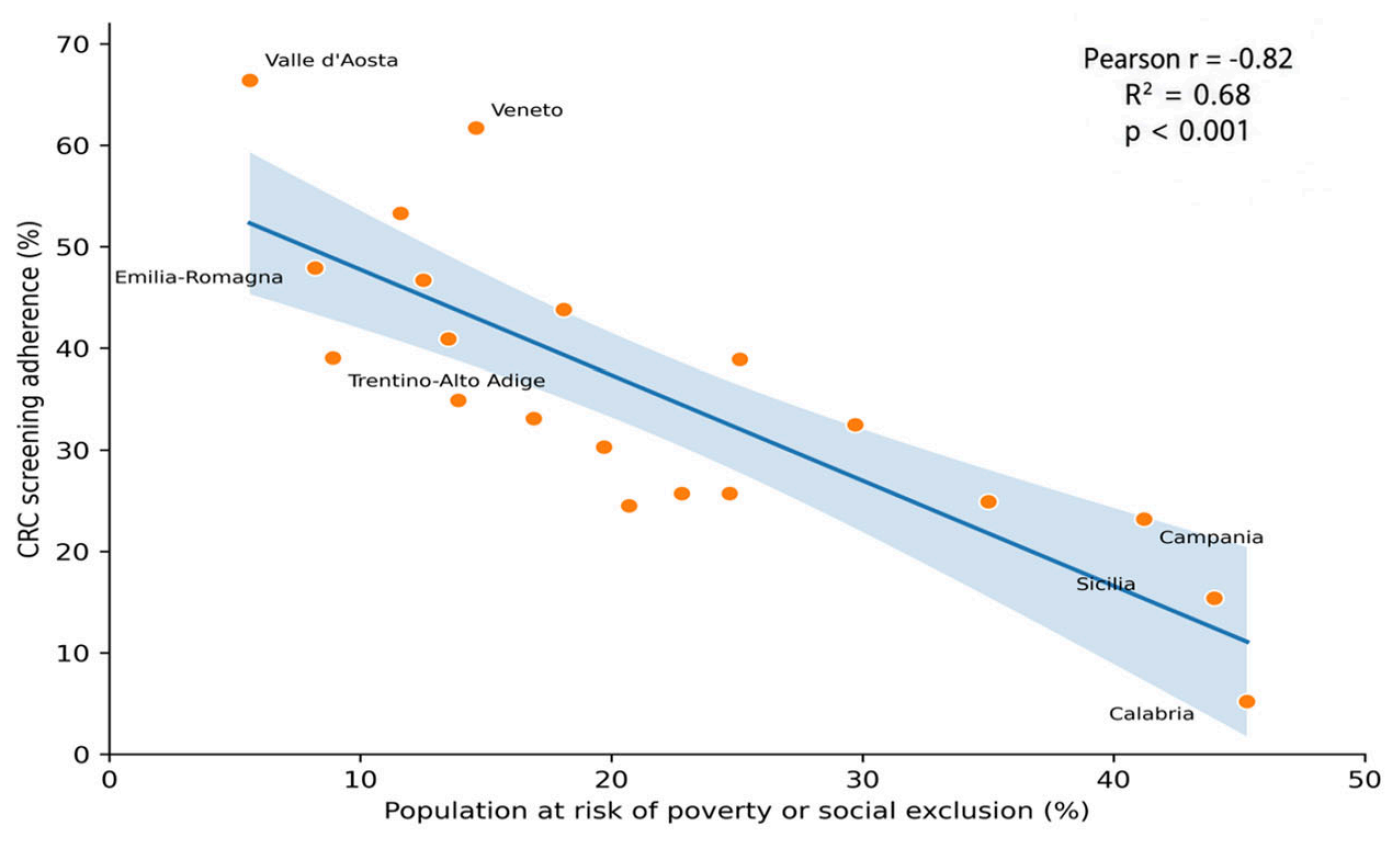
Scatterplot showing the association between population at risk of poverty or social exclusion and CRC screening adherence. The solid blue line represents the fitted linear regression, and the shaded area indicates the 95% confidence interval. Each orange circle represents one Italian regional unit included in the analysis.

Among all the evaluated indicators, the proportion of the population at risk of poverty or social exclusion demonstrated the strongest significant inverse correlation with screening uptake (Table 4). Among the evaluated indicators, the proportion of the population at risk of poverty or social exclusion showed the strongest inverse correlation (*r* = −0.781, *p* < 0.001). Similar associations were observed for low work intensity, risk of poverty, and severe material and social deprivation. Conversely, the resident-to-PCP ratio showed a moderate positive crude association with screening adherence (*r* = 0.512, *p* = 0.018), indicating that regions with greater physician workload frequently coincided with socioeconomically advantaged areas characterised by higher screening participation (Figure 2).

The four socioeconomic deprivation indicators were strongly intercorrelated, with Pearson correlation coefficients ranging from 0.79 to 0.99 (Supplementary Table S1). Variance inflation factor analysis confirmed substantial multicollinearity when all deprivation indicators were simultaneously included in the exploratory model (Supplementary Table S2). Accordingly, the final multivariable model was selected as the most parsimonious and statistically appropriate specification, including the resident-to-primary care physician ratio and the composite AROPE indicator.

To evaluate the independent contribution of primary care workforce capacity after adjustment for socioeconomic conditions, a multivariable linear regression model was fitted including the resident-to-PCP ratio and the proportion of the population at risk of poverty or social exclusion (Table 5).

The model explained 63.6% of the regional variability in CRC screening adherence (*R^2^* = 0.636; adjusted *R^2^* = 0.596; *F*(2,18) = 15.74; *p* < 0.001).

Socioeconomic deprivation remained the only variable significantly associated with CRC screening adherence after adjustment for primary care physician workload.

Each 1-percentage-point increase in the population at risk of poverty or social exclusion was associated with an estimated 0.88-percentage-point reduction in screening adherence.

After adjustment, primary care workload was no longer independently associated with screening participation (β = 0.021; 95% CI −0.017 to 0.059; *p* = 0.266). The estimated coefficients, standard errors, and overall goodness-of-fit metrics for the multivariable linear regression analysis are presented in Table 4.

**Figure 3:**
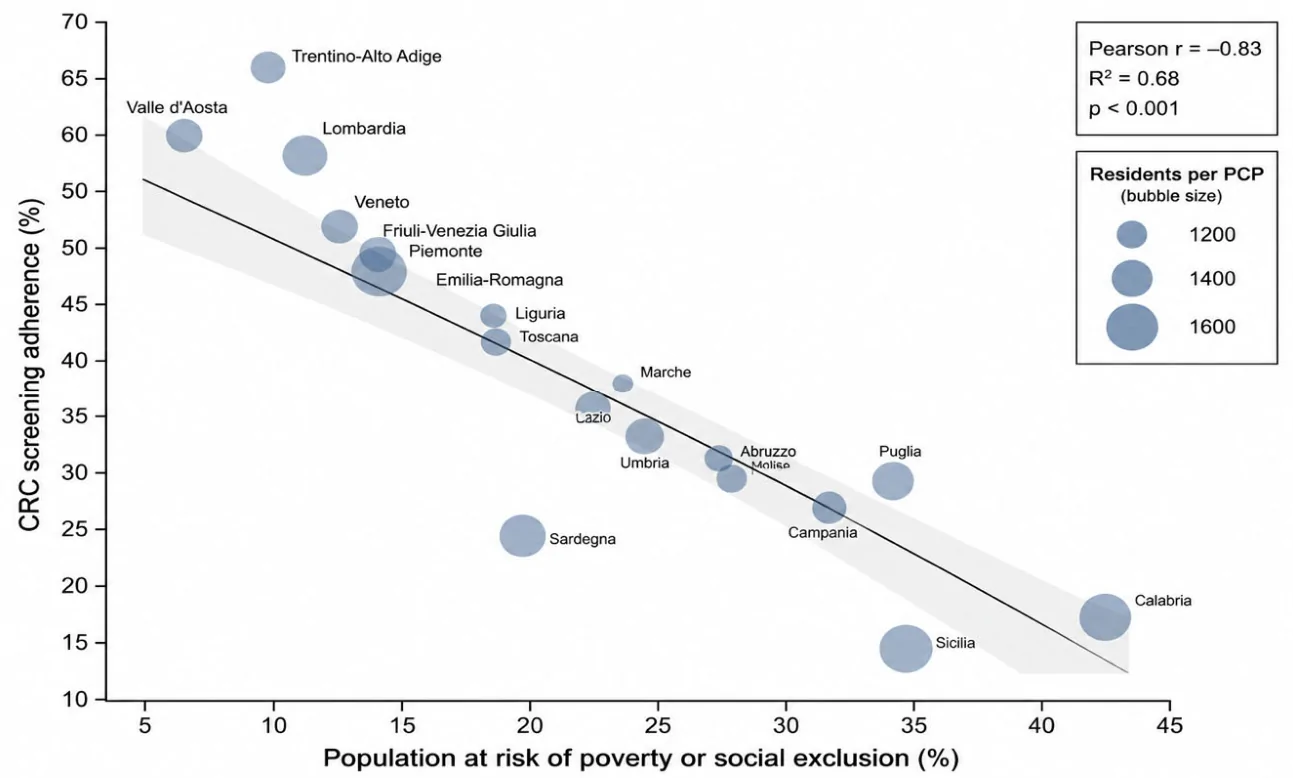
Bubble plot illustrating the relationship between socioeconomic deprivation, primary care workload (bubble size), and CRC screening adherence.

To better visualize the simultaneous impact of these factors, Figure 3 presents a bubble plot illustrating the relationship between socioeconomic deprivation and screening adherence, with the bubble size reflecting the regional density of general practitioners. Residual diagnostics confirmed the adequacy of the fitted model. Residuals were normally distributed (Shapiro–Wilk *p* = 0.752); no evidence of heteroscedasticity was observed (Breusch–Pagan *p* > 0.05). The simultaneous inclusion of all deprivation indicators produced severe multicollinearity, with VIF values ranging from 13.01 to 242.34 among the socioeconomic variables (Supplementary Table S2). By contrast, both predictors in the final model had a VIF of 1.29, indicating negligible multicollinearity.

Sensitivity analyses using alternative socioeconomic deprivation indicators yielded findings consistent with the primary analysis (Supplementary Table S3). Regardless of the specific deprivation measure included, socioeconomic disadvantage remained significantly and inversely associated with CRC screening adherence, whereas the resident-to-primary care physician ratio was not independently associated with screening participation in the primary analyses. Leave-one-region-out sensitivity analyses confirmed the robustness of the primary findings (Supplementary Table S4).

### 3.3. Spatial Autocorrelation Analysis

**Figure 4:**
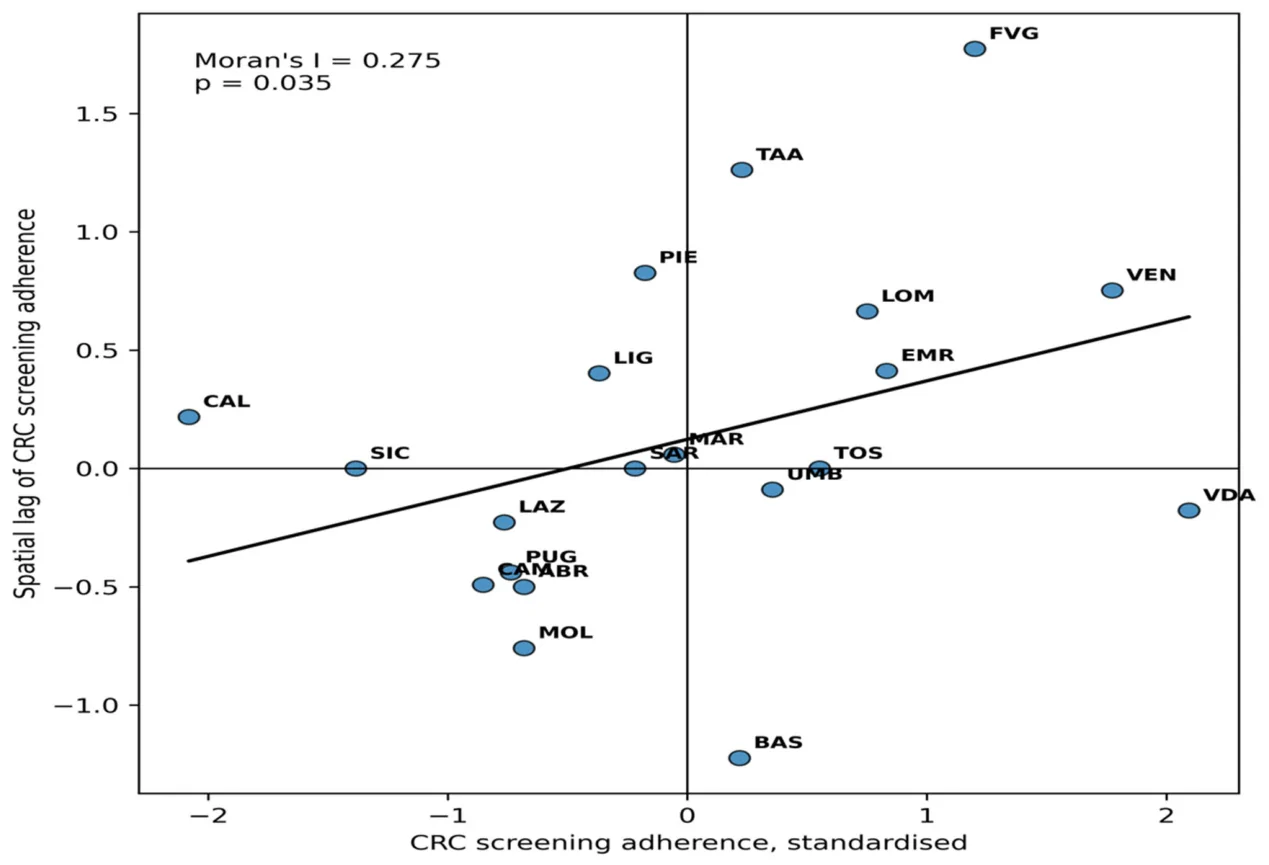
Moran scatterplot of colorectal cancer screening adherence across Italian regions.

Global Moran’s I indicated significant positive spatial autocorrelation in CRC screening adherence (I = 0.275; permutation *p* = 0.039), suggesting that neighbouring regions tended to exhibit similar screening participation levels (Figure 4).

**Figure 5:**
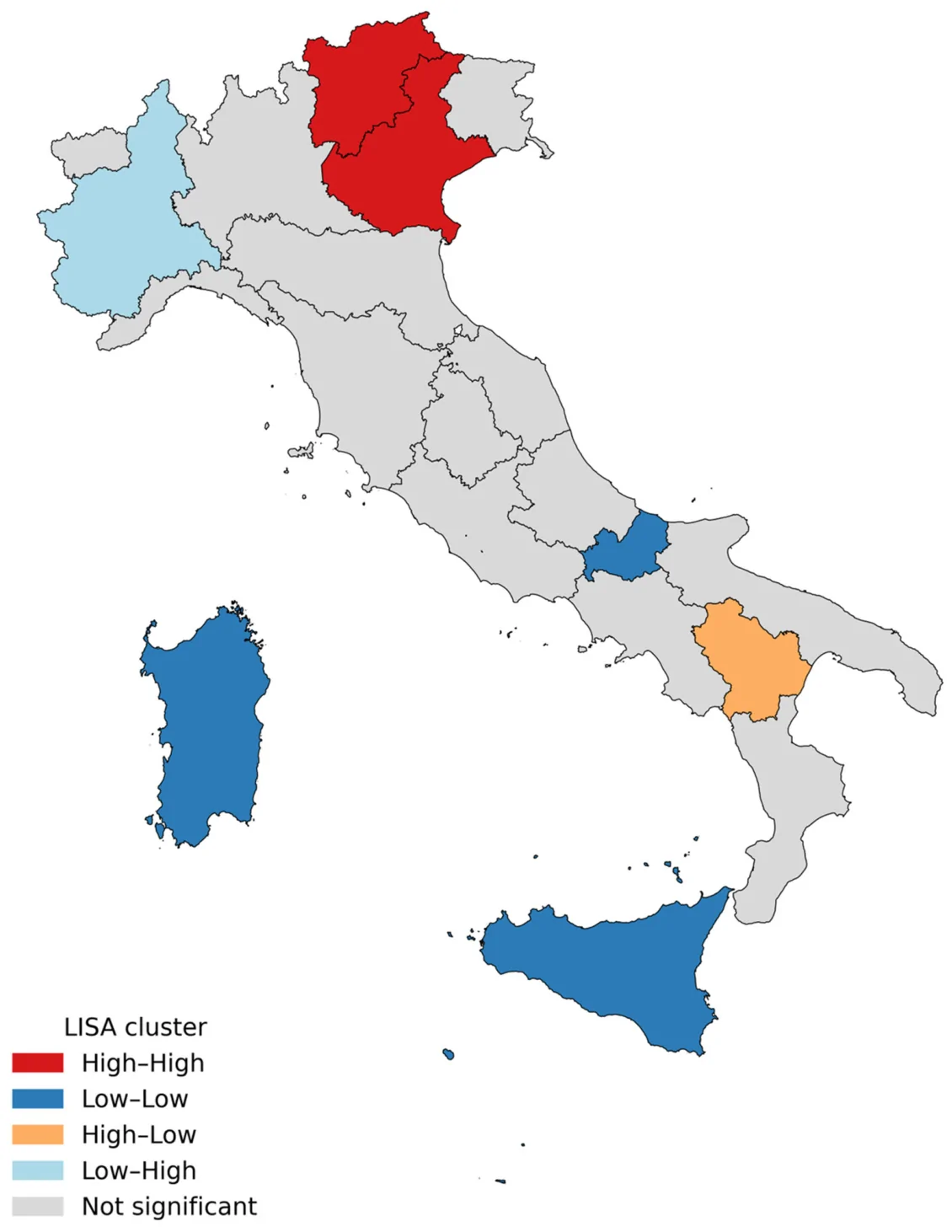
Local Indicators of Spatial Association (LISA) cluster map of colorectal cancer screening adherence in Italy.

Local Indicators of Spatial Association (LISA) identified significant High–High clusters in Northern Italy and Low–Low clusters in Southern and insular regions, together with a limited number of spatial outliers (Figure 5). However, no significant spatial autocorrelation was detected in the residuals of the multivariable ordinary least squares (OLS) regression model (*I* = −0.190, two-sided permutation *p* = 0.317), indicating that the included covariates adequately accounted for the observed spatial structure and supporting the appropriateness of the non-spatial OLS model.

Most Italian regions did not demonstrate statistically significant local spatial autocorrelation, suggesting that spatial dependence was concentrated within a limited number of regional clusters rather than being uniformly distributed across the country.

## 4. Discussion

This study investigated the relationship between organised colorectal cancer (CRC) screening participation, primary care workforce capacity and socioeconomic deprivation across the Italian regions. Three main findings emerged. First, substantial geographic inequalities in CRC screening adherence persist throughout Italy, with a clear north–south gradient. Second, socioeconomic deprivation was strongly and inversely associated with screening participation. Third, although primary care workload was correlated with screening uptake in univariate analyses, its independent contribution disappeared after adjustment for socioeconomic deprivation, suggesting that social determinants represent the principal ecological drivers of regional inequalities in screening participation.

The spatial analyses further complement these findings. Although only a limited number of statistically significant clusters were identified, Moran’s I demonstrated that CRC screening adherence is not randomly distributed across Italy. Rather, neighbouring regions tend to share similar screening performance, suggesting that regional organisation of healthcare services, socioeconomic context and common administrative practices contribute to geographically structured screening behaviour. The identification of High–High and Low–Low clusters indicates that interventions aimed at improving screening uptake may benefit from a geographically targeted approach rather than isolated regional initiatives.

The marked regional variability observed in our study is consistent with previous national reports and international evidence showing considerable heterogeneity in the implementation and effectiveness of organised CRC screening programmes [22]. Northern Italian regions generally achieved substantially higher participation rates than southern regions, reflecting longstanding differences in healthcare organisation, preventive service delivery and population engagement [23]. These findings reinforce previous observations that equal availability of organised screening programmes does not necessarily translate into equitable utilisation. The positive crude association between the resident-to-PCP ratio and screening adherence should not be interpreted as evidence that greater physician workload improves screening participation. Rather, it reflects ecological confounding, because several socioeconomically advantaged northern regions simultaneously exhibited relatively high resident-to-PCP ratios and high screening adherence. Consistent with this interpretation, the association was no longer statistically significant after adjustment for socioeconomic deprivation. The robustness of the observed association was supported by multiple sensitivity analyses. Similar findings were obtained when the composite AROPE indicator was replaced by alternative deprivation measures, and the association remained stable following sequential exclusion of each regional unit. These analyses strengthen confidence that the main findings were not driven by a single socioeconomic indicator or by any individual region. Similar socioeconomic gradients have also been reported in several European organised colorectal cancer screening programmes. Analyses from the European Health Interview Survey and studies conducted in countries including Denmark, the Netherlands, and the United Kingdom have consistently shown lower screening participation among individuals with greater socioeconomic disadvantage, lower educational attainment, and reduced health literacy [24,25,26]. These findings suggest that the inequalities observed in Italy are not unique but reflect a broader European pattern in which social disadvantage remains a major barrier to equitable participation in preventive healthcare.

The strongest finding of this study is the robust inverse association between socioeconomic deprivation and CRC screening adherence. Regions characterised by higher proportions of individuals at risk of poverty or social exclusion consistently exhibited lower participation rates [27]. This relationship remained statistically significant after accounting for primary care workforce capacity, indicating that socioeconomic disadvantage exerts an independent influence on preventive healthcare utilisation. Although these factors were not directly measured in the present study, several mechanisms may explain this association, including lower health literacy, reduced awareness of cancer prevention, competing socioeconomic priorities, limited trust in healthcare institutions and practical barriers to accessing screening services [28]. Collectively, these factors contribute to widening inequalities despite universal healthcare coverage.

Individuals living in socioeconomically disadvantaged settings may also differ in the type and quality of health information they receive [29]. Compared with more advantaged populations, they may rely more heavily on informal social networks or non-institutional information sources and may have fewer opportunities to access evidence-based preventive information through healthcare professionals, institutional communication channels, or digital health resources [30]. Although these mechanisms were not directly assessed in the present study, they may contribute to lower awareness of screening programmes and reduced participation.

Primary care physicians play a fundamental role in promoting participation in organised cancer screening programmes through patient counselling, recommendation, and facilitation of preventive care [31]. In our analysis, regions with fewer residents per primary care physician tended to report higher screening adherence, suggesting that greater availability of primary care resources may facilitate preventive interventions. However, this association was no longer statistically significant after adjustment for socioeconomic deprivation, indicating that increasing workforce capacity alone is unlikely to eliminate regional disparities if broader social inequalities remain unaddressed.

Regional differences in programme organisation should also be considered when interpreting these findings. Organised colorectal cancer screening programmes in Italy may differ with respect to invitation procedures, reminder systems, FIT distribution and return pathways, laboratory organisation, follow-up coordination, and local administrative policies [4,32,33]. These organisational characteristics may influence participation independently of socioeconomic deprivation and should be explored in future studies using more detailed regional programme data.

These findings have important implications for health policy. Strategies aimed exclusively at expanding screening invitations or increasing healthcare resources may have limited effectiveness unless accompanied by interventions specifically designed to reduce socioeconomic barriers. Tailored communication campaigns, culturally appropriate educational initiatives, community-based outreach programmes, patient navigation services and stronger involvement of primary care physicians in underserved populations may substantially improve participation among socially disadvantaged groups [34,35,36]. Addressing structural determinants of health should therefore become an integral component of organised CRC screening policies.

### 4.1. Limitations

Although our study combines nationally available administrative and socioeconomic data covering the entire Italian territory, allowing direct comparison across all regions and the integration of healthcare workforce indicators with measures of socioeconomic deprivation provides a comprehensive framework for understanding regional differences in screening participation, several limitations should be acknowledged. First, the ecological design precludes causal inference and does not allow conclusions regarding individual-level behaviour, raising the possibility of ecological fallacy. Second, the relatively small number of observational units limits statistical power and the complexity of multivariable modelling. Third, regional indicators may not fully capture substantial within-region heterogeneity in socioeconomic conditions and healthcare accessibility. Moreover, potentially relevant determinants—including educational attainment, urban–rural differences, ethnicity, immigration status, health literacy, and local organisational characteristics of screening programmes—were not available and therefore could not be incorporated into the analysis. Regional screening programmes may differ with respect to invitation strategies, FIT implementation, reminder systems, laboratory organisation and local administrative policies. These organisational characteristics were not available for analysis and may have contributed to regional variability independently of socioeconomic deprivation. Furthermore, the analysis was conducted at the level of 21 regional administrative units and 20 geographic units for spatial analyses, representing a relatively coarse territorial resolution. This aggregation may conceal substantial within-region heterogeneity in screening organisation, socioeconomic conditions, urban–rural accessibility, and population composition. The limited number of observational units also reduced statistical power, constrained the number of covariates that could be entered into the regression model, and limited the precision of local spatial statistics.

In conclusion, socioeconomic deprivation appears to be the principal determinant of regional inequalities in organised CRC screening participation in Italy, whereas primary care workforce capacity plays a secondary role once social disadvantage is considered. The observed regional clustering is consistent with previous ecological research showing that multiple health determinants—including environmental exposures, demographic ageing and healthcare-related factors—tend to concentrate within the same geographic areas [37]. Reducing disparities in CRC screening will therefore require integrated policies addressing both healthcare delivery and the broader social determinants of health. Future research should evaluate individual-level determinants of screening participation and assess the effectiveness of targeted interventions aimed at improving equity in cancer prevention.

### 4.2. Policy Implications

The present findings have important implications for the design of equitable colorectal cancer prevention strategies. Increasing screening coverage should not rely solely on expanding programme availability or healthcare workforce capacity but should also address the social determinants that influence participation. Regional screening programmes should incorporate targeted interventions for socioeconomically disadvantaged populations, including personalised invitation strategies, community-based outreach, culturally tailored educational campaigns, patient navigation services, and stronger engagement of primary care physicians in promoting screening adherence [38]. These measures are particularly relevant in the context of the European Union’s efforts to reduce inequalities in cancer prevention and to achieve higher participation rates in organised screening programmes. Integrating socioeconomic indicators into the planning and evaluation of CRC screening policies may help identify underserved populations and allocate resources more effectively [39].

## 5. Conclusions

In this ecological study, socioeconomic deprivation consistently emerged as the principal regional correlate of organised colorectal cancer screening adherence across Italy. Although primary care physician workload showed a crude association with screening participation, this association was no longer evident after adjustment for socioeconomic deprivation. Because of the ecological design, these findings should be interpreted as regional-level associations rather than evidence of causality.

Future screening policies should therefore combine interventions addressing social deprivation with geographically targeted public health strategies, particularly in areas characterised by persistent clusters of low screening uptake. Integrating spatial epidemiology into the monitoring of organised screening programmes may facilitate more efficient allocation of healthcare resources and improve equity in cancer prevention.

## Figures and Tables

**Table 1:** Operational definitions, measurement methods and data sources for study variables.

Variable	Operational Definition and Measurement	Data Source
Colorectal cancer (CRC) screening adherence (%)	Percentage of invited individuals aged 50–69 years who completed a faecal immunochemical test (FIT) within the organised colorectal cancer screening programme during the 2024–2025 screening round.	Italian National Screening Observatory (Osservatorio Nazionale Screening, ONS)
Residents per Primary Care Physician (PCP)	Calculated by dividing the resident population of each region by the number of active Primary Care Physicians. Higher values indicate a greater physician workload and lower theoretical availability for preventive care.	National Agency for Regional Health Services (AGENAS)
Population at risk of poverty or social exclusion (%) (AROPE)	Percentage of individuals experiencing at least one of the following conditions: at risk of poverty, severe material and social deprivation, or living in a household with very low work intensity, according to the Eurostat AROPE definition.	Italian National Institute of Statistics (ISTAT)
Population at risk of poverty (%)	Percentage of individuals whose equivalised disposable income is below 60% of the national median equivalised disposable income.	Italian National Institute of Statistics (ISTAT)
Severe material and social deprivation (%)	Percentage of individuals experiencing an enforced lack of at least seven of the thirteen material and social deprivation items defined by Eurostat.	Italian National Institute of Statistics (ISTAT)
Low work intensity (%)	Percentage of people aged 0–64 years living in households in which working-age members worked less than 20% of their total work potential during the previous 12 months.	Italian National Institute of Statistics (ISTAT)

Note: Definitions of the socioeconomic indicators follow the official Eurostat and ISTAT methodology. AROPE = At Risk of Poverty or Social Exclusion.

**Table 2:** Regional characteristics of organised colorectal cancer screening, primary care workforce capacity and socioeconomic indicators in Italy (2024–2025).

Region	CRC Screening Adherence (%)	Residents per PCP	Population at Risk of Poverty or Social Exclusion (%)	Population at Risk of Poverty (%)	Severe Material and Social Deprivation (%)	Low Work Intensity (%)
Piemonte	33.1	1419	16.9	14.0	3.7	4.6
Valle d’Aosta	66.4	1462	5.6	5.0	NA	NA
Lombardia	46.7	1595	12.5	9.2	4.4	3.6
P.A. Bolzano	28.6	1560	7.0	4.6	NA	NA
P.A. Trento	50.9	1413	10.8	8.8	NA	4.9
Veneto	61.7	1567	14.6	12.4	2.1	2.7
Friuli-Venezia Giulia	53.3	1566	11.6	9.1	NA	5.7
Liguria	30.3	1419	19.7	17.1	3.9	7.1
Emilia-Romagna	47.9	1446	8.2	6.7	1.7	2.1
Toscana	43.8	1474	18.1	12.8	6.4	4.7
Umbria	40.9	1242	13.5	11.3	1.8	6.5
Marche	34.9	1420	13.9	12.0	1.5	4.8
Lazio	24.5	1349	20.7	17.9	2.0	5.9
Abruzzo	25.7	1245	24.7	20.0	5.3	6.7
Molise	25.7	1170	22.8	17.5	NA	13.5
Campania	23.2	1425	41.2	35.6	10.0	20.3
Puglia	24.9	1357	35.0	27.6	9.3	13.0
Basilicata	38.9	1169	25.1	21.9	4.5	6.5
Calabria	5.2	1316	45.3	32.8	14.8	16.4
Sicilia	15.4	1199	44.0	38.4	9.4	17.1
Sardegna	32.5	1526	29.7	26.1	4.0	14.3

Note: NA = indicator not available in the official ISTAT database for the corresponding region. These values do not represent missing observations generated during the study.

**Table 3:** Descriptive statistics of study variables.

Variable	Mean ± SD	Minimum	Maximum
CRC screening adherence (%)	35.9 ± 15.1	5.2	66.4
Residents per Primary Care Physician	1397.1 ± 133.3	1169	1595
Population at risk of poverty or social exclusion (%)	21.0 ± 12.0	5.6	45.3
Population at risk of poverty (%)	17.2 ± 10.0	4.6	38.4
Severe material and social deprivation (%)	5.3 ± 3.8	1.5	14.8
Low work intensity (%)	8.4 ± 5.5	2.1	20.3

**Table 4:** Pearson correlation between study variables and organised CRC screening adherence.

Predictor	Pearson’s *r*	*p* Value
Population at risk of poverty or social exclusion	−0.781	<0.001
Low work intensity	−0.758	<0.001
Population at risk of poverty	−0.748	<0.001
Severe material and social deprivation	−0.729	0.001
Residents per Primary Care Physician	+0.512	0.018

**Table 5:** Multivariable linear regression model evaluating determinants of organised colorectal cancer screening adherence.

Predictor	B	SE	95% CI	*p* Value
Intercept	25.015	27.892	−33.583 to 83.613	0.382
Residents per Primary Care Physician	0.021	0.018	−0.017 to 0.059	0.266
Population at risk of poverty or social exclusion	−0.875	0.203	−1.303 to −0.448	<0.001

Model performance: *R^2^* = 0.636; Adjusted *R^2^* = 0.596; *F*(2,18) = 15.74; *p* < 0.001.

## Data Availability

The original contributions presented in this study are included in the article/Supplementary Material. Further inquiries can be directed to the corresponding authors.

## Supplementary Materials

The following supporting information can be downloaded at https://www.mdpi.com/article/10.3390/medsci14040436/s1, Table S1: Pairwise Pearson correlations among study variables considered for multivariable modelling; Table S2: Variance inflation factors for candidate predictors and for the final multivariable model; Table S3: Sensitivity analyses using alternative socioeconomic deprivation indicators in multivariable linear regression models of colorectal cancer screening adherence; Table S4: Leave-one-region-out sensitivity analysis of the primary multivariable regression model.
