# Supplementary material for: Socioeconomic Deprivation, Primary Care Physician Workload and Colorectal Cancer Screening Adherence Across Italian Regions: An Ecological Spatial Analysis
Source: Med Sci (Basel). 2026 Jul 26;14(4):436. doi: 10.3390/medsci14040436 (PMC13515431; doi:10.3390/medsci14040436)
Supplement: Supplementary file 1 [file medsci-14-00436-s001.zip › medsci-4449064-supplementary.pdf]

Supplementary Materials: Socioeconomic Deprivation, Primary Care Physician Workload and Colorectal Cancer Screening Adherence Across Italian Regions: An Ecological Spatial Analysis

Caterina Elisabetta Rizzo [1,*,†], Vincenzo Restivo [2,†], Maria Concetta Rizzo [3] and Cristina Genovese [4,*]

[1] Department of Prevention, Local Health Authority of Enna, 94100 Enna, Italy
[2] Department of Medicine and Surgery, University of Enna "Kore", 94100 Enna, Italy; vincenzo.restivo@unikore.it
[3] Department of Political and Social Sciences, University of Catania, 95100 Catania, Italy; maria.concetta.2000@hotmail.it
[4] Department of Biomedical Sciences, Dental Sciences and Morpho-functional Imaging, University of Messina, 98100 Messina, Italy
* Correspondence: caterina.rizzo93@gmail.com (C.E.R.); crigenovese@unime.it (C.G.)
† These authors contributed equally to this work.

Supplementary Table S1. Pairwise Pearson correlations among study variables considered for multivariable modelling.

Reference variable	Comparison variable	*n*	Pearson's *r*	*P* value
**Screening adherence**	Residents per PCP	21	0.512	0.018
	AROPE	21	−0.781	<0.001
	Population at risk of poverty	21	−0.748	<0.001
	Severe material and social deprivation	16	−0.729	0.001
	Low work intensity	19	−0.758	<0.001
**Residents per PCP**	AROPE	21	−0.472	0.031
	Population at risk of poverty	21	−0.478	0.028
	Severe material and social deprivation	16	−0.258	0.334
	Low work intensity	19	−0.359	0.131
**AROPE**	Population at risk of poverty	21	0.989	<0.001
	Severe material and social deprivation	16	0.886	<0.001
	Low work intensity	19	0.911	<0.001
**Population at risk of poverty**	Severe material and social deprivation	16	0.805	<0.001
	Low work intensity	19	0.910	<0.001
**Severe material and social deprivation**	Low work intensity	16	0.793	<0.001

Abbreviations: AROPE, *At Risk of Poverty or Social Exclusion*.

Supplementary Table S2. Variance inflation factors for candidate predictors and for the final multivariable model.

Model	Predictor	*n*	VIF
**Model including all candidate predictors**	Residents per PCP	16	1.52
	AROPE	16	242.34
	Population at risk of poverty	16	163.11
	Severe material and social deprivation	16	20.10
	Low work intensity	16	13.01
**Final multivariable model**	Residents per PCP	21	1.29
	AROPE	21	1.29

Abbreviations: AROPE, *At Risk of Poverty or Social Exclusion*; PCP, primary care physician; VIF, variance inflation factor.
Note: The model including all candidate predictors was based on complete cases; therefore, only 16 regional observations were available. The intercept was excluded from the table. VIF values greater than 5 indicate potentially important multicollinearity, while values greater than 10 indicate severe multicollinearity.

Supplementary Table S3. Sensitivity analyses using alternative socioeconomic deprivation indicators in multivariable linear regression models of colorectal cancer screening adherence

n	Socioeconomic indicator	B (95% CI)	Standardized β	*P* value	Residents per PCP B (95% CI)	Standardized β	*P* value	Adjusted R^2
**21**	Population at risk of poverty or social exclusion (AROPE)	−0.875 (−1.303 to −0.448)	−0.694	<0.001	0.021 (−0.017 to 0.059)	0.185	0.266	0.596
**21**	Population at risk of poverty	−0.990 (−1.538 to −0.442)	−0.652	0.001	0.023 (−0.018 to 0.064)	0.200	0.259	0.545
**16**	Severe material and social deprivation	−2.302 (−3.624 to −0.981)	−0.635	0.002	0.039 (0.000 to 0.079)	0.365	0.050	0.602
**19**	Low work intensity	−1.626 (−2.450 to −0.802)	−0.632	<0.001	0.037 (0.003 to 0.071)	0.351	0.033	0.642

Abbreviations: AROPE, *At Risk of Poverty or Social Exclusion*; PCP, primary care physician.
Note. Each model included the resident-to-primary care physician ratio and one socioeconomic deprivation indicator. Complete-case analysis was performed; therefore, sample size varied according to the availability of regional socioeconomic data.

Supplementary Table S4. Leave-one-region-out sensitivity analysis of the primary multivariable regression model

Excluded region	AROPE B (95% CI)	*P* value	Residents per PCP B	*P* value	Adjusted R^2
**Piemonte**	−0.886	0.001	0.021	0.273	0.605

	(−1.322 to −0.450)				
**Valle d'Aosta**	−0.773<br>(−1.192 to −0.354)	0.001	0.022	0.217	0.571
**Lombardia**	−0.875<br>(−1.317 to −0.434)	0.001	0.022	0.291	0.583
**P.A. Bolzano**	−0.961<br>(−1.315 to −0.606)	<0.001	0.030	0.067	0.742
**P.A. Trento**	−0.849<br>(−1.295 to −0.402)	0.001	0.022	0.258	0.581
**Veneto**	−0.882<br>(−1.280 to −0.485)	<0.001	0.012	0.520	0.612
**Friuli-Venezia Giulia**	−0.869<br>(−1.306 to −0.431)	0.001	0.018	0.356	0.573
**Liguria**	−0.877<br>(−1.311 to −0.442)	0.001	0.021	0.264	0.604
**Emilia-Romagna**	−0.877<br>(−1.331 to −0.422)	0.001	0.021	0.281	0.580
**Toscana**	−0.876<br>(−1.316 to −0.436)	0.001	0.020	0.302	0.591
**Umbria**	−0.862<br>(−1.327 to −0.397)	0.001	0.022	0.285	0.592
**Marche**	−0.898<br>(−1.335 to −0.461)	<0.001	0.021	0.281	0.609
**Lazio**	−0.887<br>(−1.313 to −0.461)	<0.001	0.019	0.313	0.611
**Abruzzo**	−0.881<br>(−1.321 to −0.440)	0.001	0.019	0.343	0.588
**Molise**	−0.893<br>(−1.339 to −0.446)	0.001	0.017	0.421	0.588
**Campania**	−0.932<br>(−1.426 to −0.437)	0.001	0.018	0.365	0.584
**Puglia**	−0.888<br>(−1.346 to −0.431)	0.001	0.021	0.287	0.583
**Basilicata**	−0.840<br>(−1.263 to −0.416)	0.001	0.032	0.123	0.633
**Calabria**	−0.777<br>(−1.261 to −0.293)	0.004	0.023	0.234	0.506
**Sicilia**	−0.908<br>(−1.375 to −0.442)	0.001	0.022	0.255	0.555
**Sardegna**	−0.887<br>(−1.352 to −0.422)	0.001	0.020	0.336	0.593

Note. The primary multivariable model was repeated after excluding one region at a time. The association between AROPE and CRC screening adherence remained statistically significant in all iterations, whereas the resident-to-primary care physician ratio remained non-significant throughout.
